# Demineralized Dentine

**Published:** 1876-08

**Authors:** J. E. Cravens

**Affiliations:** Indianapolis, Ind.


### ARTICLE II.

### *Demineralized Dentine.*

BY J. E. CRAVENS, INDIANAPOLIS, IND.

In the *Dental Register*, October No., 1872, page 412, there was published a letter from me on the use, by dentists, of the Syrup of Lacto-Phosphate of Lime.

It was the first of the various epistles I have written upon this solution of bone phosphate, and in which I advocated the use of it internally as a tonic calculated to benefit patients suffering from an absence of the mineral constituents of tooth-bone proper. At that time the medical profession had just begun prescribing this syrup to such of their patients as were suffering from diseases causing a deficiency of the prime earth element of the osseous system, or owing to which that condition was liable to eventuate.

In the letter referred to, I advocated the administration of the Syrup form of Lacto-Phosphate of Lime by dentists to patients whose teeth were manifestly deficient as to inorganic elements, be the cause whatsoever it might. It is my purpose here to furnish a few instances from my own practice in support of the views I then advanced, and to renew the advocacy of this character of tonic treatment.

It is not infrequently the case that a Miss of ten or twelve years, while presenting no symptoms of any particular disease, nevertheless painfully impresses the observer with her peculiar physical appearance. She is very thin and pale, while her movements are languid and indicate an absence of energy. She is weak at all times, her appetite is scant and fickle, whilst sleep is indulged in, or rather submitted to as a matter of duty, and is unrefreshing to her. The girl is, in fact, delicate, and there would seem to be an arrest of development generally, were it not that she grows, grows rapidly, too—not out, but up, like Jonah's gourd, so to speak.

This young lady is quite sure to find her way to the dentist. Should *he* know what practice to pursue with her peculiar case, that *thorns will disappear*, and her pathway to his office will be strewn with roses. If, to the contrary, the dentist falls short of the proper treatment of the patient's infirmity, her *teeth* will disappear, and the ugly thorns of regret and impaired digestion will hedge her earthly pil grimage.

The teeth of this patient are of that bluish-white or pearl-starch complexion that the intelligent practitioner of dentistry dislikes so much to have placed under his care. Visibly the cavities are small, more or less numerous, and for the main part confined to the side teeth. A thorough examination with a small pointed excavator about the grinding surface lines of the molars, develops wretched condition of them —*sous enamel.* The intercuspidal grooves that mark the junctions of the folds of original enamel membrane may appear to be defective to only the extent usual in those lines. But the deceptiveness of such teeth is revealed when the instrument passes directly through the enamel fissure into the body of the crown, and is revolved around and around, meeting with little or no opposition. The crown is a mere shell, a veritable whited sepulchre, and full of rottenness.

It is an easy matter to cave in the edges of the enamel fissure overhanging the cavern; and having done so, and removed the chips, we see—what? A tray of soft fibrous debris that emits a vile odor. The fibrous mass is demineralized dentine; the intertubular substance having been dissolved away, the cartilaginous tubuli have possibly enlarged, or their walls thickened from saturation, therefore the fibres. Particles of the softer food have been forced through the fissures by the opposing tooth or teeth in mastication; then the saliva having gained ingress a partial digestion ensues; the natural heat of the parts soon induces a sort of garbage fermentation—decomposition—hence the odor.

The character of dental caries in such cases is that known as the *soft white.* Under the soft or fibrous mass the dentine has at first a cheese like consistency, and is usually exasperatingly sensitive, although the density improves as the excavator advances. When the instrument uncovers dentine that has not been subjected to the action of the contents of the cavity of caries, it will be noticed that the cartilaginous or organic element predominates in the ivory structure, and that an exalted sensibility still prevails, and the verdict is necessarily *demineralization.*

It is not my intention to give here in my views of the proper *topical* treatment of a tooth affected as above described, because, hypothetically, if one tooth has suffered from a general structural loss of inorganic constituents, all the teeth in that mouth have experienced the same character of inanition, and probably to a corresponding degree.

In 1872 a girl of 12 years was brought to my office for treatment of her teeth. She was of the description I have just given; indeed it has been my aim to illustrate, with a pen-picture of this young lady, that peculiar physical debility and dentinal starvation, the systemic treatment of which I hope to elucidate in this paper. This girl did not obtain puberty uutil two years after my treatment, so that her peculiar physical frailness could hardly be attributed to the approaching first change in female life. When she was placed under my charge she had then been suffering four years from the debility and weakness described, during which time she had been under homœopathic treatment but had failed to obtain any relief. I directed her to take pulverulent phosphate of lime, two tea-spoonfuls daily, one morning and one evening. In about a week she called to say that the powder was becoming disagreeable to her, and requested that the number or size of daily doses be diminished. I told her to take thereafter one spoonful a day and that in the morning. In one week more her father called to say that his daughter could not swallow the powder at all, and that she declared she could even smell it, her aversion to it having gradually increased from the outset, so that finally the sight of it caused a sickening revulsion. On examining the powder she had, I found nothing wrong about it. It was white, clear and odorless; so that I was induced to think that her stomach having been unable to digest the powder, phosphate of lime, the repeated introduction of it into the gastric cavity superinduced a dyspeptic, morbid mental impression that was immediately aggravated through the sense of sight. The action on the olfactory nerve was imaginary, and probably owing to the peculiar sympathy existing between the special senses of sight, taste and smell. I directed a discontinuance of the powder that in two weeks had become so obnoxious to the patient.

About this time I read M. Locke's report of the successful experiments in certain French hospitals, with lacto-phosphate of lime, the phosphate having been predigested in lactic acid, and then prepared with a syrup vehicle to be administered to patients.

The marvelous beneficial effects of this lacto solution of bone phosphate were obtained through the stomach, and therefore the pabulum must have been generally distributed throughout the osseous structures of the body; even the teeth were not debarred from the gracious banquet. Imagine the pleasure of Quartermaster Pulp upon receiving such wholesome supplies for distribution to his hungry little regiment.

When I read the wonders detailed in M. Locke's article, I hoped I had at last found the desired remedy for the troubles of my young patient. At my suggestion her father procured four ounces of the syrup, and gave it to her in dessert-spoon doses, three times per diem, before or after meals; the doses were after a time reduced to two, and finally to one spoonful daily.

From the first of this syrup treatment the girl began to improve. Her appetite increased rapidly and permanently, her cheeks began to show some color, whilst her eyes brightened and her spirits became light and joyous. Strength and roundness returned to her frame, that had formerly been languid, weak and wasted.

When the first supply of this tonic was exhausted her parents renewed it; the patient continued to improve in every respect, so that the mother thought to make an extended visit, accompanied by her daughter, to Massachusetts, where the family formerly resided. The mother had not been able to revisit the scenes of her earlier life since moving west, several years before, because the feeble health of her daughter would not admit of it. Before starting upon their journey the mother and daughter called at my office, that I might see the wonderfully improved condition of the young lady—to have her teeth examined, and any temporary stoppings renewed that were needed—to show me a fresh supply of the lacto-phosphate of lime, purchased to "tide" the patient over the proposed absence, and to say "good-bye" for about four months.

About the end of the allotted time the patient returned, and soon visited my office. Her general rotundity was certainly delightful to those who had known and pitied the emaciated, fading flower of but a few months previous. Her formerly thin face was round and rosy, and her efforts to shape her mouth into a broad smile soon ended in a well-marked dimple in either cheek. I freely confess that the great physical improvement of the patient under the syrup tonic was not contemplated by me at the outset, and my astonishment was fully equal to that of the parents. I did not say this to them, however, but received their expressions of gratitude in dignified silence.

But to return to the consideration of the effects of this treatment upon the patient's teeth. When placed under my charge the extreme sensitiveness and softness of the dentine prevented me from introducing more than two or three gold fillings, so that I stopped the remaining cavities temporarily, to await the good effects hoped from the lacto-phosphate of lime. Some of these temporary fillings come out, and some I removed at various times, affording me ample opportunity to note any changes in sensibility of the dentine. Very soon an improvement in this respect was noticeable, becoming gradually more marked until the patient left on the four months' visit east. After her return I found the sensitiveness of dentine had subsided to a normal state, and I removed most of the temporary stoppings and refilled with gold. The patient said she felt no aversion toward the syrup from first to last, but on the contrary she rather liked it because it tasted like gooseberry juice and sugar. I must dismiss this patient.

In the early fall of 1871, Mrs. ———, a lady of twenty-six years, being pregnant, I persuaded her to an experiment course with phosphate of lime, so that in early gestation she began taking the powder, one or two tea-spoons of it daily. This she practiced faithfully until about the sixth month, when I substituted the syrup of lacto-phosphate of lime. During the time that she was taking the dry phosphate she was plump, rosy-checked and strong ; no nausea, no headaches or backaches; her appetite was generally good, and she never felt averse to swallowing the powder. Then she began, at the sixth or seventh month to take the syrup, her appetite increased rapidly until she ate ravenously. She said she really was always hungry, but had to stop eating occasionally, for politeness' sake, and a due regard for her husband's exchequer. She felt perfectly well until labor claimed her place ; the pains were of short duration, and the child was a boy of ten pounds; he was a lusty-voiced little fellow, and his flesh remarkably solid, feeling and looking like that of a child several months old. This boy is now nearly four years old, large, and unusually strong for the age, and a picture of health. He has a full set of deciduous teeth, good, strong and regular, with plenty of room in the jaws. The mother has given birth to another boy since then; the weight at birth was also ten pounds; but the child's flesh was softer, and he was not so vigorous as his elder brother. The same difference obtains between these boys to-day, for, although the younger one is about two years old, he looks like a delicate little girl, his skin is very clear and white, his voice more like a girl's than a boy's, his stature small, and whilst apparently fleshy, his weight is deceptively light. During the second gestatory period, the mother had received no phosphate of lime, except as it was derived from food; her appetite was good enough perhaps under the circumstances, but she was much troubled with headache, pains in the lumbosacral region, and occasional sense of nausea.

Whilst pregnant with the first child, and during the succeeding lactation, the mother experienced no unusual trouble from her teeth, and having been called upon during this time to introduce one or two fillings, I took care to notice the condition of the dentine, and found no unusual sensibility there.

The second gestation and ensuing lactation, however, afforded quite a diverse report. Shortly after this second impregnation, the lady's teeth began to trouble her; they continually broke away in bits, and were painfully susceptible to thermal changes. Withal, her nervousness so increased that she could not endure a dental operation at all, even though it were painless. After her babe had been weaned she was again strong enough to take her place in my operating chair, but her dental apparatus was hopelessly wrecked. The deduction in the case of this mother and her two boys is that the demand of the fœtal development upon the mother's system was too great for her to fulfill, unless her own self were helped to an additional supply from external sources, and in greater abundance than was afforded by ordinary alimentation. In the case of the first gestation the mother received an abundance of the bone phosphate, and was accordingly enabled to give of her store sufficient to develop strong bones and muscular fibres in the fœtus, without suffering any appreciable loss in her own osseous system. But in the second gestatory period how different were the results. The life intrauterine was developed at the expense of the tissues of the mother. The food she ate did not supply the requisite phosphate of lime, so that her bones could spare but little to the fœtal development. Nevertheless, the bones—teeth and all—were robbed to the up-building of the little stranger, and for want of the external resources from which to obtain the needed pabulum re mained, robbed and softened, until lactation ceased, and the babe no longer derived nourishment from the mother.

I report only the foregoing cases because they are marked instances of the beneficial effects to be obtained from tonic treatment of our patients with lacto-phosphate of lime in cases where for any reason the teeth are deficient in mineral constituents. I have prescribed this syrup in many cases with good effects from the use of it, and I believe that if the profession would oftener resort to this manner of dealing with subjects afflicted with general dentinal hyperæsthesia, previous to or during numerous painful operations, and where soft white dental caries prevails, they would find their operations more satisfactory to themselves, would benefit humanity, and receive the heartfelt gratitude of their patients.—*Missouri Dental Journal.*

